# Are Measures of Health Status for the Total Population Good Proxies for the Health of the Older Population in International Comparison Studies?

**DOI:** 10.3390/ijerph19137559

**Published:** 2022-06-21

**Authors:** Ewa Kocot, Sabina Denkowska, Kamil Fijorek

**Affiliations:** 1 Health Economics and Social Security Department, Institute of Public Health, Faculty of Health Sciences, Jagiellonian University Medical College, 31-066 Krakow, Poland; 2 Department of Statistics, Institute of Quantitative Methods in Social Sciences, College of Economics, Finance and Law, Cracow University of Economics, 31-510 Krakow, Poland; sabina.denkowska@uek.krakow.pl (S.D.); kamil.fijorek@uek.krakow.pl (K.F.)

**Keywords:** population health, health indicators, older population, comparative analyses

## Abstract

In the face of population aging, the health of older people is becoming especially important, impacting various areas of life, societies and countries’ economies. To provide the basis for effective decisions to achieve better health, comparative analyses can be used to find best practices to follow. The aim of the research was to check whether drawing conclusions about the older population’s health based on the health status of the total population is justified in international comparison analyses. An analysis was conducted for six population health indicators for European countries from 2010–2019. Rankings were created for the total population and the older subpopulation, and then ranks for these two populations were compared using statistical methods. The statistical analyses indicate that there is a strong, statistically significant relationship between the ranks for the total and the older population. However, looking at the descriptive analysis and visual presentation of data, differences in international rankings of indicator values for these two populations can be observed. As older people comprise a specific group of the population that is growing ever bigger and increasingly significant, it would be advisable to present the results of international comparisons not only for the total, but separately for the older population as well.

## 1. Introduction

Improving population health and reducing health inequalities are goals of every society, regardless of its current health status level. To ensure a solid basis for making the right decisions to achieve better population health, it should be measured and monitored constantly, using valid, well-constructed indicators. Monitoring health status helps allocate healthcare resources in an adequate way and assess the results of public health activities. Health indicators of population health are used for making comparisons between countries and benchmarking, as well as assessing changes over time within countries [1].

Comparative health analyses were conducted as long ago as the 17th century, in the area of hospital mortality. In the 1990s, the comparison has been structured as a method of different aspects of healthcare analyses [2,3]. Comparisons carried out at different levels and in settings (e.g., regional, international, between hospitals, etc.) make it possible to identify disparities, analyze the reasons for them, and find the best practices possible to improve various aspects of healthcare quality. International comparisons of population health using health-related indicators (including summary measures) make an important contribution to the evaluation of health system performance and enable policymakers to learn from others, helping to direct policy in the right direction [4,5,6]. International comparisons help identify high performers and provide policymakers with a benchmark in order to identify areas that are functioning below expectations [7,8].

Any time comparison results or ranking lists are published, especially if they are international and related to health or healthcare, it causes a huge wave of comments and even protests. The comparison results are broadly covered in the media, as issues related to health always attract widespread attention [4]. Prime examples include the global debate that started after the WHO had announced its health-system performance assessment in 2000, and the comments that have appeared in the media each time the Health Consumer Powerhouse publishes a new Euro Health Consumer Index report [7,9,10].

The rankings of population health, or those in which population health is one of the components, often use values for the total population, not presenting information for any subgroups (for example, the Bloomberg Global Health Index [11], the OECD Better Life Index [12], and the Global Health Security Index Ranking [13]). Meanwhile, older people constitute a growing and more significant group in many populations. In the last 20 years (from 2001 to 2021), the size of the population 65 and above in European Union (EU) countries has increased by over 37% and the share of this population in the total EU population has risen from 15.8% to 20.8% [14]. People who are older contribute in many ways to societies, and the expectations for the social participation of older groups are increasing, but these opportunities are highly dependent on older people’s health [15,16]. In most EU countries the group of people from 15 to 64 years old (that is, of the defined working age) is shrinking: in 16 countries, the size of this group declined between 2001 and 2021, even by nearly 25% in Latvia [14]. As a result, the older population group is more and more important in the formal and informal labor force [16]. To plan and implement appropriate actions to ensure healthy and active aging, it is necessary to identify and assess older people’s health status and functioning [15]. Measuring, monitoring, and understanding the older population’s health is crucial to ensure the best possible functional ability and to build a sufficient long-term care system [17]. The question arises of whether a comparison analysis using total population health indicators is sufficient in the presented context.

The research question of the study was whether drawing conclusions about older population health based on the health status of the total population is justified in international comparison analyses. To answer this question, international comparisons between European countries were taken into account. A review of population health status indicators available in international databases was also conducted and the construction of these indicators was checked in the context of the older-age population group.

## 2. Materials and Methods

A review of the five biggest international open-access databases was conducted (Eurostat [14], WHO Global Health Observatory (WHO GHO) [18], Global Health Data Exchange (GHDx) [19], OECD Data [20], World Bank Open Data [21]) to identify population health status indicators and gather available values. These databases contain a wide range of health-data collections and declare a statistics comparability, which is one of the essential features in international analyses. The necessity of data comparability was the main reason for relying on international databases, and not on national sources. As a sample for the study, 27 European Union countries + the United Kingdom, Norway, Switzerland and Iceland were chosen. The main rationale for this choice was data availability in the Eurostat database, which is the only one that contains some indicators for assessing population health that are not available elsewhere. 

Finally, six indicators were chosen for the analysis: life expectancy (LE), self-perceived health (SPH), healthy life expectancy based on self-perceived health (HLE), healthy life years (HLY), healthy life expectancy (HALE), and disability-adjusted life years (DALY) (Table 1). The analysis covers the period of 2010–2019, except for the HALE indicator, for which the analysis was carried out for the years 2010, 2015 and 2019 due to the lack of data availability.

For all selected indicators and for each year from 2010–2019, the rankings were created for both the total population and the older subpopulation, and then ranks for these two populations were compared. A higher indicator value means a higher ranking position, excluding DALY, for which a lower value means a higher position (as DALY indicates a burden of diseases). When indicator values are equal, their ranks are the same and equal to the arithmetic mean of their “potential” positions.

The statistical analysis contains the descriptive statistics and then more advanced statistical analyses. Because rankings are an example of ordinal-type data, nonparametric statistical tests were used for the analysis. The sign test and the Wilcoxon signed-rank test were applied to compare the distributions of ranks for the total population and the older subpopulation. The strength of the relationship was assessed using Spearman’s and Kendall’s rank correlation coefficients (the detailed description of methods used in the analysis can be found in the Supplementary Materials S1). While the sign test is based on the number of signs of differences, the Wilcoxon test takes into account the magnitude of differences in pairs by considering the absolute rank values of these differences. Therefore, the Wilcoxon test is considered more powerful than the sign test.

For all indicators, linear regression models of the dependence of the ranks for the older subpopulation on the ranks for the total population for 2010–2019 were also estimated. The resulting regression coefficient indicates how much the rank of the older subpopulation will increase on average if the rank of the whole population increases by one position. In turn, the coefficient of determination $\left(R^{2}\right)\;$ indicates what proportion of the variation in ranks for the older subpopulation is predictable from the ranks for the total population, and the root-mean-square error (RMSE) shows the mean deviation between the observed values and the values predicted by a model. The statistical analyses were performed in STATA. 

## 3. Results

### 3.1. Indicators of Population Health

**Figure 1:**
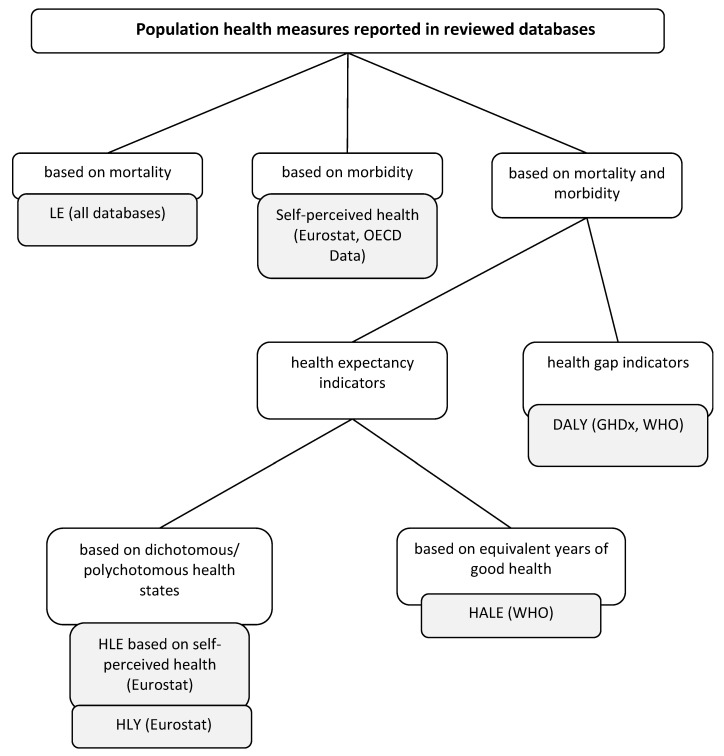
The general classification of population health status measures. Note: LE—life expectancy; DALY—disability-adjusted life years; HLE—healthy life expectancy based on self-perceived health; HLY—healthy life years; HALE—healthy life expectancy. Source: own work based on [6].

The indicators selected for the analysis cover all of the main categories of the measures applied to measure population health (the adopted framework can be found in Figure 1).

#### 3.1.1. Life Expectancy (LE)

Life expectancy is a widely used popular measure of population health. Its values are reported by all main databases and international institutions. One of the important shortcomings of this indicator is the fact that it does not take into account health status directly, using only mortality as a proxy. Despite this, LE is still used in many analyses, including international comparisons, as mortality data are routinely collected by countries from a variety of sources and interpretation of differences in these indicator values is intuitive [22,23,24,25]. 

Life expectancy at a given age (in this analysis, at birth and at age 65) represents the number of years that a person at that age is expected to live. In most cases (including those presented in the accessed databases), it is calculated on the assumption that age-specific mortality levels remain constant in the future: thus, period life expectancy is used (the method of indicator calculation is presented in detail in the Supplementary Materials S2). This means that death probability at each age is assumed to be the same, regardless of the cohort for which LE is calculated. For example, the same value is used for probability of death at age 70 for LE at birth (that is, concerning an event in 70 years) and LE at age 65 (an event in 5 years). Due to the fact that LE is based on the current values of age-related mortality, indicators for which LE is a component can be taken as measures of the current health of the total population when calculated “at birth.” Any changes in mortality rates (and in LE and as a consequence) for the older population strongly influence changes of LE at birth (and at any younger age), as all values used in calculations for the older age group are included in calculations for every younger group, and the probability of death is much higher in older age groups than in younger. In the case of countries with low mortality in the younger population (such as in Europe), decreasing mortality rates at older ages is one of the main reasons for a rise in LE [16,26].

#### 3.1.2. Self-Perceived Health (SPH)

Self-perceived health is a single-item measure of health-related quality of life, expressing an entirely subjective evaluation of health in general. It indicates overall perception of respondents’ health, including both physical and psychological dimensions [27]. Although this indicator is very simple in construction, in many cases it may be a good predictor of morbidity and mortality [28,29,30,31]. The source of the self-perceived health data presented in the Eurostat database is The European Statistics of Income and Living Conditions (EU-SILC) survey. The question asked to the respondents is: “How is your health in general? Is it…” with the possible answers: very good/good/fair/bad/very bad [32]. The results are presented as a share of the given group of people who gave each of the responses, for various age groups. The results for each separate age group are independent in a given year, but for a total population are dependent on the answers for each group included in it (including the group 65+). However, this impact on the results for the entire population is more significant in the case of larger groups and less significant with smaller ones. As the population of people 65+ in European countries accounts for 14.6% to 23.5% of the total population—and in more than half of the countries is lower than 20% (2021 data) [14]—the impact of this group’s health on the total health assessment may be expected to be relatively small. However, as the share of the older population is steadily growing (according to the Eurostat baseline projection, the share of 65+ population in the total population will increase in the EU 27 from 20.8% in 2021 to nearly 30% in 2050, by as much as 13 percentage points in Spain [14]), the health of the older group will affect the value for the total population more and more (a more detailed explanation is provided in the Supplementary Materials S2).

#### 3.1.3. Health Expectancy Indicators

Health expectancy indicators combine data regarding mortality and morbidity, giving information on how many years a person is expected to live in a given state of health if current mortality and morbidity conditions continue to apply. This group of indicators expands the idea of life expectancy by adding a component related to the prevalence of nonfatal health outcomes. In the indicator construction, (1) dichotomous or polychotomous health states or (2) equivalent years of good health can be defined. Depending on the adopted definition of health, a health expectancy indicator can firstly estimate for a group the average number of years that a person could expect to live, e.g., without disability, in a good self-perceived health, without functional limitations; or in a case of the second definition, group indicator health states are weighted according to health problem severity [6,33]. Three indicators of the general health expectancy group were identified for the analysis: healthy life expectancy based on self-perceived health (HLE), healthy life years (HLY), and healthy life expectancy (HALE). The first two represent measurement using dichotomous states of health, and the latter represents measurement indicating the expectation of equivalent years of good health (more information about indicator construction can be found in the Supplementary Materials S2). As the general idea of health expectancy indicators is to combine life expectancy (as described above) with issues connected with morbidity, their value depends on both fractions. 

##### Healthy Life Expectancy Based on Self-Perceived Health (HLE) and Healthy Life Years (HLY)

The HLE and HLY indicators are based on a directly determined, subjective self-assessment of health. In the first, the self-perceived health indicator value (as described above) is used as a measure of good and bad health; in the second one, the opinion regards long-standing activity limitations. It is natural that both bad self-perceived health and activity limitations occur much more frequently in older age. This means that the gap between a general LE value and the expectancy of living in health is caused to a greater extent by health deterioration in older groups of people than in younger groups.

In the European Union in 2020, 19.1% people over 65 years old declared bad or very bad health (these health states are taken into account as lowering health expectancy in HLE calculation), while it was only 4.9% in the 16–64 age group [14]. Looking at activity limitation data (used in HLY calculation) for the European Union in 2020, only 51% of people of age 65+ did not declare any limitations, while 83% people younger than 65 years did [14]. Therefore, in both cases, the values for older people can be expected to have a substantial influence on the total.

In health expectancy measures using dichotomous health valuation (as HLY and HLE) two health states are used (defined according to indicators): good and bad. Years lived in the bad state are valued at zero (equivalent to death), and years in the good state at one (equivalent to full health). This means that these measures are not sensitive to differences in severity “inside” each of the two health states (internal variety), which may be significant. However, the indicator value is highly sensitive to health-state differences around the threshold separating good health from bad [6]. As on average the health status of younger people is quite good, years for this group are mostly considered to be equal to full health, even if they are not indeed perfectly healthy. This may be a reason for the overestimation of a total population health. Counting every heath state below a given threshold as zero, even if it is not as bad as death (mostly the case in the older population) may strongly decrease the value and cause underestimation.

##### Healthy Life Expectancy (HALE)

In the HALE estimation, a wide group of health states is used, which are weighted according to severity. As continuous health-state valuation is used, the problem with under- and overestimation due to “internal variety” does not influence results significantly.

Life-expectancy trends are mainly driven by prevalence rates [34]. As prevalence rates are mostly higher in older age, their values are very important for the total value. In the simpler form of HALE adopted some time ago by the WHO, age-weighting is not applied [35]. In the age-weighting procedure, the assumption was that life years lived at different ages should be valued differently, and as a result, less weight was given to years of healthy life lost at younger and older ages [6]. Abandoning the age-weighting process in calculating HALE means that older people’s health problems have a stronger impact on the total HALE value.

#### 3.1.4. Health Gap Indicator: Disability-Adjusted Life Years (DALY)

The DALY measure presents time lost due to health problems, in contrast to the previously described indicators, which presented a “positive” approach (time expected to live in a good health). As the prevalence of health problems is much higher in the older population and DALY indicates the burden of diseases, values for the total population are strongly affected by the values for the older population. In low-mortality countries, such as in Europe, most of the burden of disease is concentrated at older ages [6]. The total burden of disease in DALYs (all causes) for people 70+ ranged in EU countries from 33% in Slovakia to 47.1% in Italy, while the population at age 70 and above was only 10.2% and 17.1%, respectively [14,19].

As with the HALE indicator, the age-weighting procedure stopped being used some time ago. Due to this decision, the total DALY number is significantly higher now and the share of DALYs for the older and younger population has increased [36]. Switching the DALY calculation to the prevalence-based approach (having previously been incidence-based) changed the age distribution of YLDs, e.g., in the case of noncommunicable diseases, the value for younger groups decreased, and for older groups increased substantially [36]. As noncommunicable diseases account for over 80% of total DALYs [19], the importance of prevalence in the older population for the overall value is much more significant now.

### 3.2. Descriptive Statistics

Table 2 presents information the share of countries with rank differences for the total and for the older population.

The share of countries with a ranking-position difference depends on the year and indicator type, with values ranging from 67.7% (HLE in 2015) to 100% (LE and HLY in 2015), but in the vast majority, it exceeds 80% (41 out of 53).

Table 3 provides descriptive statistics for indicator rank differences between the older subpopulation and the total population from 2010 to 2019 (additionally, the analysis of rank quotients is presented in the Supplementary Materials S3). Large variations in difference values can be observed between both indicators and years. Looking only at the absolute values of differences (meaning not taking into account which population is ranked higher: total or older) it can be observed that in the case of one indicator (HLY), maximum absolute differences are not lower than 10 over the entire analyzed period. For two indicators (HLE and HALE), the absolute values of the maximum rank difference are always less than 10, and for the remaining ones, there are values both greater and lower than 10 (depending on the year).

The largest SD of the LE rank difference was observed in 2011 (4.18), with the highest maximum (Cyprus’s LE rank for the older subpopulation was 19.5 and for the total population was 9) and absolute minimum (Finland’s LE rank for the older subpopulation was 9.5 and for the total population was 18.5 in 2011) values. In the year 2019, the lowest SD for an LE indicator was observed, with absolute differences not higher than 5 (equal to 5 for Cyprus and −5 for France). The highest SD of the SPH rank difference was observed in 2018, but the highest value of maximum (14 for Cyprus) and absolute value of minimum (10 for UK) were in 2016. In the case of HLE, the highest maximum value (9) was observed in 2014 (Cyprus) and in 2017 (Italy), the highest absolute minimum value was in 2010 (5.5 for Denmark), but the largest SD was 2.92, in 2019. The HLY indicator was characterized by high SD values and rank differences. The range of SD values was from 4.79 (in 2010 and 2016) to 6.26 (in 2014) and the rank differences even exceeded 15 (for Greece in 2013). For HALE indicator, there were only three years with available data, but in these years rank difference values were not high, with absolute values of differences from 4.0 (for the Netherlands in 2015) to 6.5 (for Norway in 2010) and SD from 2.25 to 2.69. The largest SD of the DALY rank difference was observed in 2012 (5.09), but the highest maximum value (12) was in 2010–2013 and the highest absolute minimum value (9) was in 2011–2012. In general, looking at all indicators and all years, the biggest rank difference was observed in 2013 for Sweden (HLY, 15.5) and the highest SD for HLY was in 2014 (6.26).

In the entire analysis period, and for all indicators, the rank differences did not exceed five positions for the vast majority of countries (see Table S2 in Supplementary Materials). Only in 39 cases was this difference higher than 10, of which 32 were differences in the rankings related to SPH and HLY. The rankings for each indicator and year are also presented in a graphical form in the Supplementary Materials S5. Based on this presentation, the biggest discrepancies between the total and the older population are clearly seen for two indicators: SPH and HLY.

### 3.3. Advanced Statistical Analysis

#### 3.3.1. The Sign Test and the Wilcoxon Test; Spearman’s Correlation and Kendall’s Correlation

The results of the sign test and the Wilcoxon test for the analyzed indicators in years 2010–2019 are presented in Table 4, Table 5, Table 6, Table 7, Table 8 and Table 9. For all indicators between 2010 and 2019, we fail to reject the null hypothesis of the equality of the distributions of the total population and the older subpopulation. Both Spearman’s test and Kendall’s test [37,38,39,40] indicate a strong, statistically significant relationship between ranks for the total population and ranks for the older subpopulation over the entire period of 2010–2019 (2010, 2015 and 2019 for HALE).

#### 3.3.2. Regression Analysis

The rank analysis was supplemented by a regression model of the dependence of the indicator ranks for the older subpopulation on the indicator ranks for the whole population from 2010 to 2019. The results presented in Table 10 confirm the statistically significant relationship between the ranks for the total population and the older subpopulation for all analyzed indicators.

The regression coefficient indicates that an increase of one rank in an indicator ranking for the total population means an average increase in the ranking for the older subpopulation of at least 0.8 ranks. For four of the analyzed indicators this coefficient is 0.9 or more, with the highest value for two of them (HLE and HALE, both equal to 0.96). Only HLY and SPH are characterized by a coefficient value lower than 0.9 (0.81 and 0.87, respectively).

The share of rank variability for the older population explained by rank variability for the whole population varies depending on the indicator. Only about 66% of HLY rank variability for the older population is explained by HLY rank variability for the total population, but for HLE and HALE it is as much as 93%. The second-lowest result is observed for SPH at 75%. The remaining scores are 81% for DALY and 89% for LE.

The root-mean-square error (RMSE) shows that the mean deviation between the observed values and those predicted by the model is in the range of 2.44 to just over five. The lowest values of RMSE are observed for HLE (2.44) and HALE (2.46). The worst result among the considered indicators occurs in the case of HLY and is as high as 5.12.

## 4. Discussion

The aim of the research was to evaluate whether drawing conclusions about older population health based on the health status of the total population is justified in international comparison analyses. To do this, rankings of countries in terms of the health status of the total and the older population were compared. The analysis was conducted for 31 European countries, and six indicators were used to evaluate population health for the period of 2010–2019.

The statistical analysis did not reject the null hypothesis of equality of the distributions of the total population and the older subpopulation for any of the analyzed indicators (this does not mean that this distributions’ equality has been proven, but we cannot state that it does not exist). The tests also indicate that there is a strong, statistically significant relationship between ranks for the total and the older population. However, looking at the descriptive analysis and visual presentation of data, differences in international rankings of indicator values can be noted. The ranking position of the total population rarely differs from that of the older population by more than 10 items, but sometimes this difference can reach even 15 positions. Thus, in individual cases, the difference is considerable and may affect the conclusions of the ranking analysis. The highest rank differences can be observed for the HLY indicator, and in each year of the analysis period, the SD of rank differences for HLY is higher than 4.7. This value is also exceeded for half of the analyzed years for the SPH indicator, but it occurs very rarely for the remaining ones (only three years for DALY). This suggests that the answer to the research question may depend to some extent on the kind of indicator used, and the analyzing rankings for both total and older populations may be more important for some indicators than for others. The regression analysis also indicates that conclusions drawn from the rankings for the total and the older population may differ more for HLY than, for example, for HLE and HALE; as for the HLY rank variability, for the older group it is explained by the rank variability for the total population only in 66% of cases, while for the last two it is 93%.

International comparisons can be a good measure for identifying potential improvements, but a deep understanding of the reasons for differences between a given country and the best performer is needed [8]. Due to ranking differences, even when not statistically significant, another country (or countries) can be indicated as examples of “best practices,” looking at total population health and at the older population ranking. As a result, health policy decisions regarding older population health may be based on policies of a country with even worse health outcomes. Looking at health population assessments only through the prism of the entire population may lead to conclusions that do not fully take into account the needs of subpopulations, including older people. For example, in 2019 the HLY indicator ranking for the total population of Cyprus was 10 places higher than Denmark, but in the ranking for the older population, it was 14 places lower. This suggests that analysis regarding the reasons for these differences should be conducted separately for the total and the older populations.

The population health indicator rankings are presented not only as a single comparison, but often constitute one of the components of summary indices in complex assessment frameworks. They may regard not only health-system performance or healthcare-quality evaluation, but also assessments of a wider scope, such as, for example, the Sustainable Development Goals Index, which includes, i.a., life expectancy at birth [41]. In this index a health indicator for the total population was used, just like in many other cases (Bloomberg Global Health Index [11], OECD Better Life Index [12], Global Health Security Index Ranking [13], and the World Health Report index [9]), but a good example of the deliberate use of an indicator for the older instead the total population is the Health Care System Performance Rankings published by the Commonwealth Fund. The authors indicate that they are assessing health-system performance, so measures should reflect outcomes that can be modified by health care, while life expectancy at birth may be affected more by socioeconomic conditions than by health-system activities [42].

Health indicators applicable to all age groups are not easy to construct. Measures evaluating activity limitations may be good for examining the older population’s health status, but may not be good or sensitive enough for the total population [43]. Measures based on self-perceived health are not used for children, and, for example, in the case of HLY and HLE, the health status of the youngest population is estimated based on the answers of the first interviewed age group, which can impact assessment reliability (assumed to be half of the prevalence for the group 16–19) [44]. There is also a problem in using these measures for people who, due to their health state, cannot consciously fill questionnaires in (mostly in the older age groups). In HALE construction, disability weights are applied that reflect the severity of a given disease/injury outcome. However, using the same disability weights for every age group may be questionable, as health is perceived differently at different ages. The acceptable level of a health state usually becomes lower with age, so assessment of the burden caused by health problems may be very age-dependent. A health problem greatly lowering a young person’s quality of life can be seen as not so important by an older person. However, an opposite difference is also possible—older people can start to see good health as more important and pay more attention to health [45,46,47,48,49]. This may indicate that slightly different methods should be used to measure the health of the older population, or at least that the indicators should be computed partially, paying attention to different age groups [15,43]. Meanwhile, there is still a lack of scientific consensus on the key indicators for creating proper health policy and public health interventions for older people [50].

Population health indicators are often used in international comparisons, sometimes in combination with other kind of measures, to analyze relationships between health and various factors. Some good examples are studies on the relation between GDP and health (e.g., [51,52]). Preparing such analyses, especially when their results may have an impact on policy decisions regarding not only the total but also the older population specifically, it is worth checking whether conclusions would be similar if not the total but rather the older population health were taken into account. This would ensure that the decision was being made on a reliable basis.

Regardless of the choice of health indicator, the value for the older population affects more or less the value for the total population. Looking at indicator construction, it may be supposed that this impact is quite strong, but only a detailed analysis could show how significant this impact is depending on the indicator. This kind of analysis would be worth conducting in the future.

This study has been prepared for a small group of countries, all of them situated in Europe. These are countries of a relatively similar level of population health, which may be considered a limitation of the research. A similar analysis should be conducted for other, non-European countries to check the results. Due to data-availability issues, in the case of two indicators, the older population is defined as something other than 65 and over: 60 for HALE and 70 for DALY. This inconsistency can also be seen as a limitation. Additionally, HALE indicator data are only available in the analyzed period for three years.

The results of this research indicate that there are no statistically significant differences between total and older population health assessment in international comparisons. Regardless, despite the lack of statistical confirmation, some differences in EU country rankings may be observed, and they can in some cases affect the conclusions drawn from benchmarking analyses. Every ranking, especially international ones, arouses great interest and emotions. Additionally, taking into account differences in health perception, assessment, expectations and needs in older and younger age groups, it seems justified to recommend presenting the results of population health comparisons not only for the total population, but for the older population as well, at least if it is used as the basis for planning specific health policy and interventions aimed at older people. Data regarding the older population’s health are still insufficient, not systematically collected, analyzed or interpreted [50].

## 5. Conclusions

International comparisons and different kinds of analyses based on benchmarking may help policymakers make the best decisions regarding actions to improve population health. Although the statistical analysis of indicator values indicates the strong relationship between health status rankings for the total and the older population, the differences that may be important for health policy decisions are observed. As older people are a specific group of the population that is still growing and is increasingly significant in society, it would be advisable to present the results of international comparisons not only for the total, but separately for the older population as well. The introduction of modified indicators for the older population should be also considered.

## Figures and Tables

**Table 1:** Indicators chosen for the analysis.

Indicator Name	Indicator for Total Population	Indicator for Older Population	Data Source
Life expectancy (LE)	Life expectancy in absolute value at birth, in years	Life expectancy in absolute value at 65, in years	Eurostat [14]
Self-perceived health (SPH)	Self-perceived health for age 16 and over, very good or good, in percentage	Self-perceived health for age 65 and over, very good or good, in percentage	Eurostat [14]
Healthy life expectancy based on self-perceived health (HLE)	Health expectancy based on self-perceived health in absolute value at birth, in years	Health expectancy based on self-perceived health in absolute value at 65, in years	Eurostat [14]
Healthy life years (HLY)	Healthy life years in absolute value at birth, in years	Healthy life years in absolute value at 65, in years	Eurostat [14]
Healthy life expectancy (HALE)	Health-adjusted life expectancy at birth, in years	Health-adjusted life expectancy at age 60, in years	WHO GHO [18]
Disability-adjusted life years (DALY)	DALYs age-adjusted per 100,000 population, all ages, in DALY	DALYs age-adjusted per 100,000 population, 70 years and over, in DALY	GHDx [19]

**Table 2:** Share of countries with rank differences for the total and the older population.

Year	LE		SPH		HLE		HLY		HALE		DALY	
	*n*	%	*n*	%	*n*	%	*n*	%	*n*	%	*n*	%
2010	30	90.0	31	93.5	31	80.6	30	86.7	31	77.4	31	87.1
2011	31	96.8	31	90.3	31	71.0	31	80.6	N/A	N/A	31	90.3
2012	30	93.3	31	87.1	31	74.2	30	90.0	N/A	N/A	31	90.3
2013	30	93.3	31	93.5	31	80.6	30	90.0	N/A	N/A	31	93.5
2014	31	93.5	31	77.4	31	71.0	31	96.8	N/A	N/A	31	87.1
2015	31	100.0	31	77.4	31	67.7	31	100.0	31	83.9	31	87.1
2016	31	80.6	31	77.4	31	80.6	31	96.8	N/A	N/A	31	90.3
2017	31	87.1	31	87.1	31	83.9	31	87.1	N/A	N/A	31	80.6
2018	31	96.8	31	77.4	31	77.4	31	90.3	N/A	N/A	31	80.6
2019	30	86.7	29	79.3	29	82.8	29	96.6	31	83.9	31	74.2

Note: n denotes number of analyzed countries; LE—life expectancy; SPH—self-perceived health; HLE—healthy life expectancy based on self-perceived health; HLY—healthy life years; HAL—healthy life expectancy; DALY—disability-adjusted life years.

**Table 3:** Descriptive statistics for rank differences between the older population and the total population 2010–2019.

Indic.	Year	*n*	SD	Min	Max	Indic.	Year	*n*	SD	Min	Max
LE	2010	30	3.17	−7.5	7.0	HLY	2010	30	4.79	−12.0	14.0
	2011	31	4.18	−9.0	10.5		2011	31	4.87	−13.0	11.5
	2012	30	3.57	−8.0	9.5		2012	30	5.18	−13.0	11.0
	2013	30	2.58	−5.5	5.0		2013	30	5.39	−13.0	15.5
	2014	31	3.05	−5.5	10.0		2014	31	6.26	−12.5	14.0
	2015	31	2.85	−5.0	9.0		2015	31	5.50	−14.0	14.0
	2016	31	2.91	−4.5	7.0		2016	31	4.79	−10.5	11.0
	2017	31	2.93	−6.5	8.5		2017	31	5.79	−12.0	12.0
	2018	31	2.54	−5.5	6.0		2018	31	6.04	−10.5	14.5
	2019	30	2.33	−5.0	5.0		2019	29	5.65	−15.0	10.0
SPH	2010	31	4.03	−9.0	8.0	HALE	2010	31	2.53	−6.0	6.5
	2011	31	3.86	−7.0	9.0		2011	N/A	N/A	N/A	N/A
	2012	31	4.58	−9.0	12.0		2012	N/A	N/A	N/A	N/A
	2013	31	4.53	−9.0	11.0		2013	N/A	N/A	N/A	N/A
	2014	31	4.24	−8.0	12.0		2014	N/A	N/A	N/A	N/A
	2015	31	4.73	−8.0	12.0		2015	31	2.25	−4.0	5.0
	2016	31	5.17	−10.0	14.0		2016	N/A	N/A	N/A	N/A
	2017	31	4.91	−8.0	13.5		2017	N/A	N/A	N/A	N/A
	2018	31	5.34	−8.0	13.0		2018	N/A	N/A	N/A	N/A
	2019	29	4.94	−9.0	12.0		2019	31	2.69	−6.0	6.0
HLE	2010	31	2.53	−5.5	7.0	DALY	2010	31	4.88	−7.0	12.0
	2011	31	1.71	−4.0	3.5		2011	31	5.08	−9.0	12.0
	2012	31	2.12	−4.0	6.5		2012	31	5.09	−9.0	12.0
	2013	31	2.14	−3.5	7.0		2013	31	4.63	−8.0	12.0
	2014	31	2.74	−4.5	9.0		2014	31	4.07	−7.0	11.0
	2015	31	2.19	−3.5	6.0		2015	31	3.64	−7.0	7.0
	2016	31	2.85	−5.0	8.0		2016	31	3.61	−6.0	7.0
	2017	31	2.85	−5.0	9.0		2017	31	3.44	−6.0	6.0
	2018	31	2.67	−5.0	6.5		2018	31	3.11	−6.0	5.0
	2019	29	2.92	−4.0	8.0		2019	31	2.94	−6.0	5.0

Note: n denotes number of analyzed countries; SD—standard deviation; LE—life expectancy; SPH—self-perceived health; HLE—healthy life expectancy based on self-perceived health; HLY—healthy life years; HALE—healthy life expectancy; DALY—disability-adjusted life years; N/A—not applicable.

**Table 4:** Results of the sign test, the Wilcoxon test and Spearman’s and Kendall’s correlation tests for LE ranks for the total population and the older subpopulation in 2010–2019.

Year	n	The Sign Test	The Wilcoxon Test	Spearman’s Correlation	Kendall’s Correlation			
		$n-n_{0}\;\left(n_{+}\right)$	Z	$r_{s}$	$\tau_{a}$	$\tau_{b}$	S	$SE{\;}^{§}$ of S
2010	30	27 (15) †	−0.134 †	0.935 ***	0.793 ***	0.812	345	55.843
2011	31	30 (17) †	−0.137 †	0.894 ***	0.725 ***	0.742	337	58.613
2012	30	28 (14) †	−0.052 †	0.918 ***	0.752 ***	0.767	327	55.870
2013	30	28 (16) †	−0.206 †	0.957 ***	0.830 ***	0.844	361	55.908
2014	31	29 (15) †	0.403 †	0.943 ***	0.809 ***	0.838	376	58.437
2015	31	31 (16) †	−0.020 †	0.951 ***	0.822 ***	0.839	382	58.647
2016	31	27 (14) †	0.344 †	0.949 ***	0.822 ***	0.843	382	58.614
2017	31	27 (15) †	−0.029 †	0.948 ***	0.822 ***	0.835	382	58.698
2018	31	30 (17) †	0.030 †	0.961 ***	0.850 ***	0.864	395	58.695
2019	30	26 (13) †	−0.021 †	0.965 ***	0.851 ***	0.869	370	55.873

Note: n  denotes number of observations, $n_{0}$ denotes number of ties, $n_{+}$ denotes number of positive signs (+), Z denotes test statistic for Wilcoxon test. S denotes score for Kendall’s test. ^§^ means “correction for ties” of standard errors. *** denotes p < 0.001 and † denotes *p* > 0.1.

**Table 5:** Results of the sign test, Wilcoxon test and Spearman’s and Kendall’s correlation tests for SPH ranks for the total population and the older subpopulation in 2010–2019.

Year	n	The Sign Test	The Wilcoxon Test	Spearman’s Correlation	Kendall’s Correlation			
		$n-n_{0}\;\left(n_{+}\right)$	Z	$r_{s}$	$\tau_{a}$	$\tau_{b}$	S	$SE{\;}^{§}$ of S
2010	31	29 (16) †	−0.177 †	0.902 ***	0.744 ***	0.745	346	58.827
2011	31	28 (15) †	0.157 †	0.910 ***	0.755 ***	0.755	351	58.836
2012	31	27 (14) †	0.187 †	0.873 ***	0.710 ***	0.710	330	58.827
2013	31	29 (16) †	0.265 †	0.876 ***	0.723 ***	0.723	336	58.827
2014	31	24 (12) †	0.247 †	0.891 ***	0.753 ***	0.754	350	58.827
2015	31	24 (13) †	0.296 †	0.865 ***	0.723 ***	0.725	336	58.805
2016	31	24 (13) †	0.345 †	0.838 ***	0.708 ***	0.711	329	58.802
2017	31	27 (14) †	0.472 †	0.854 ***	0.710 ***	0.710	330	58.827
2018	31	24 (14) †	0.415 †	0.827 ***	0.695 ***	0.696	323	58.819
2019	29	23 (14) †	0.446 †	0.832 ***	0.687 ***	0.690	279	53.282

Note: n  denotes number of observations, $n_{0}$ denotes number of ties, $n_{+}$ denotes number of positive signs (+), Z denotes test statistic for Wilcoxon test. S denotes score for Kendall’s test. ^§^ means “correction for ties” of standard errors. *** denotes p < 0.001 and † denotes *p* > 0.1.

**Table 6:** Results of the sign test, the Wilcoxon test, and Spearman’s and Kendall’s correlation tests for HLE ranks for the total population and the older sub-population in 2010–2019.

Year	n	The Sign Test	The Wilcoxon Test	Spearman’s Correlation	Kendall’s Correlation			
		$n-n_{0}\;\left(n_{+}\right)$	Z	$r_{s}$	$\tau_{a}$	$\tau_{b}$	S	$SE{\;}^{§}$ of S
2010	31	25 (14) †	0.484 †	0.961 ***	0.858 ***	0.862	399	58.802
2011	31	22 (12) †	−0.169 †	0.982 ***	0.905 ***	0.911	421	58.785
2012	31	23 (14) †	0.624 †	0.973 ***	0.882 ***	0.886	410	58.794
2013	31	25 (15) †	0.624 †	0.972 ***	0.886 ***	0.891	412	58.794
2014	31	22 (14) †	0.876 †	0.955 ***	0.845 ***	0.849	393	58.802
2015	31	21 (11) †	0.450 †	0.971 ***	0.893 ***	0.898	415	58.785
2016	31	25 (14) †	0.435 †	0.951 ***	0.845 ***	0.852	393	58.763
2017	31	26 (16) †	0.690 †	0.951 ***	0.845 ***	0.851	393	58.785
2018	31	26 (15) †	0.542 †	0.957 ***	0.858 ***	0.862	399	58.802
2019	29	24 (14) †	0.554 †	0.941 ***	0.825 ***	0.830	335	53.264

Note: n  denotes number of observations, $n_{0}$ denotes number of ties, $n_{+}$ denotes number of positive signs (+), Z denotes test statistic for Wilcoxon test. S denotes score for Kendall’s test. ^§^ means “correction for ties” of standard errors. *** denotes p < 0.001 and † denotes *p* > 0.1.

**Table 7:** Results of the sign test, Wilcoxon test and Spearman’s and Kendall’s correlation tests for HLY ranks for the total population and the older subpopulation in 2010–2019.

Year	n	The Sign Test	The Wilcoxon Test	Spearman’s Correlation	Kendall’s Correlation			
		$n-n_{0}\;\left(n_{+}\right)$	Z	$r_{s}$	$\tau_{a}$	$\tau_{b}$	S	$SE^{\;§}$ of S
2010	30	26 (15) †	−0.320 †	0.852 ***	0.692 ***	0.697	301	55.997
2011	31	25 (13) †	−0.168 †	0.857 ***	0.677 ***	0.682	315	58.779
2012	30	27 (15) †	−0.175 †	0.827 ***	0.660 ***	0.664	287	55.997
2013	30	27 (14) †	−0.031 †	0.813 ***	0.644 ***	0.647	280	56.006
2014	31	30 (16) †	0.000 †	0.763 ***	0.581 ***	0.585	270	58.771
2015	31	31 (16) †	0.079 †	0.817 ***	0.658 ***	0.666	306	58.737
2016	31	30 (16) †	−0.049 †	0.861 ***	0.686 ***	0.689	319	58.802
2017	31	27 (14) †	−0.108 †	0.797 ***	0.613 ***	0.614	285	58.819
2018	31	28 (14) †	0.059 †	0.779 ***	0.609 ***	0.613	283	58.785
2019	29	28 (15) †	−0.335 †	0.780 ***	0.611 ***	0.615	248	53.254

Note: n  denotes number of observations, $n_{0}$ denotes number of ties, $n_{+}$ denotes number of positive signs (+), Z denotes test statistic for Wilcoxon test. S denotes score for Kendall’s test. ^§^ means “correction for ties” of standard errors. *** denotes p < 0.001 and † denotes *p* > 0.1.

**Table 8:** Results of the sign test, Wilcoxon test and Spearman and Kendall correlation tests for HALE ranks for the total population and the older subpopulation in 2010, 2015 and 2019.

Year	n	The Sign Test	The Wilcoxon Test	Spearman’s Correlation	Kendall’s Correlation			
		$n-n_{0}\;\left(n_{+}\right)$	Z	$r_{s}$	$\tau_{a}$	$\tau_{b}$	S	$SE{\;}^{§}$ of S
2010	31	24 (12) †	−0.010 †	0.961 ***	0.839 ***	0.841	390	58.810
2015	31	26 (14) †	0.108 †	0.969 ***	0.852 ***	0.854	396	58.810
2019	31	26 (13) †	0.020 †	0.956 ***	0.830 ***	0.833	386	58.810

Note: n denotes number of observations, $n_{0}$ denotes number of ties, $n_{+}$ denotes number of positive signs (+), Z denotes test statistic for Wilcoxon test. S denotes score for Kendall’s test. ^§^ means “correction for ties” of standard errors. *** denotes p < 0.001 and † denotes *p* > 0.1.

**Table 9:** Results of the sign test, the Wilcoxon test and Spearman’s and Kendall’s correlation tests for DALY ranks for the total population and the older subpopulation in 2010–2019.

**Year**	n	The Sign Test	The Wilcoxon Test	Spearman’s Correlation	Kendall’s Correlation			
		$n-n_{0}\;\left(n_{+}\right)$	Z	$r_{s}$	$\tau_{a}$	$\tau_{b}$	S	$SE{\;}^{§}$ of S
2010	31	27 (15) †	0.404 †	0.856 ***	0.669 ***	0.669	311	58.836
2011	31	28 (15) †	0.236 †	0.844 ***	0.639 ***	0.639	297	58.836
2012	31	28 (14) †	0.196 †	0.844 ***	0.656 ***	0.656	305	58.836
2013	31	29 (15) †	0.207 †	0.871 ***	0.686 ***	0.686	319	58.836
2014	31	27 (15) †	0.275 †	0.900 ***	0.742 ***	0.742	345	58.836
2015	31	27 (15) †	0.187 †	0.920 ***	0.772 ***	0.772	359	58.836
2016	31	28 (16) †	0.167 †	0.921 ***	0.772 ***	0.772	359	58.836
2017	31	25 (13) †	0.069 †	0.929 ***	0.781 ***	0.781	363	58.836
2018	31	25 (13) †	−0.020 †	0.942 ***	0.811 ***	0.811	377	58.836
2019	31	23 (12) †	−0.069 †	0.948 ***	0.819 ***	0.819	381	58.836

Note: n  denotes number of observations, $n_{0}$ denotes number of ties, $n_{+}$ denotes number of positive signs (+), Z denotes test statistic for Wilcoxon test. S denotes score for Kendall’s test. ^§^ means “correction for ties” of standard errors. *** denotes p < 0.001 and † denotes *p* > 0.1.

**Table 10:** Regression models of the dependence of the indicator ranks for the older subpopulation on the indicator ranks for the total population 2010–2019.

Indic.	Variable	Coefficient	Standard Error	Number of Obs.	$R^{2}$	RMSE
LE	rank_of_LE	0.94 ***	0.02	306	0.89	2.97
	const	0.93 **	0.35			
SPH	rank_of_SPH	0.87 ***	0.03	308	0.75	4.44
	const	2.10 ***	0.52			
HLE	rank_of_HLE	0.96 ***	0.02	308	0.93	2.44
	const	0.61 *	0.29			
HLY	rank_of_HLY	0.81 ***	0.03	305	0.66	5.12
	const	2.92 ***	0.60			
HALE	rank_of_HALE	0.96 ***	0.03	93	0.93	2.46
	const	0.61 †	0.52			
DALY	rank_of_DALY	0.90 ***	0.03	310	0.81	3.96
	const	1.64 ***	0.46			

Note: ***, **, and * indicate significance at the 0.001, 0.01, and 0.1 levels respectively and † denotes *p* > 0.1. LE—life expectancy; SPH—self-perceived health; HLE—healthy life expectancy based on self-perceived health; HLY—healthy life years; HALE—healthy life expectancy; DALY—disability-adjusted life years.

## Data Availability

The data used in the analysis not included in the article or Supplementary Materials are available from the corresponding author upon request.

## Supplementary Materials

The following supporting information can be downloaded at: https://www.mdpi.com/article/10.3390/ijerph19137559/s1: S1: The description of statistical methods; S2: The methods of the indicator calculation; S3: The analysis of quotients; S4 (Table S2): Number of countries with a given size of ranking difference; S5: The graphical presentations of rankings.
