# Supplementary material for: Are Measures of Health Status for the Total Population Good Proxies for the Health of the Older Population in International Comparison Studies?
Source: Int J Environ Res Public Health. 2022 Jun 21;19(13):7559. doi: 10.3390/ijerph19137559 (PMC9265627; doi:10.3390/ijerph19137559)
Supplement: Supplementary file 1 [file ijerph-19-07559-s001.zip › ijerph-1725928-supplementary.pdf]

SUPPLEMENTARY MATERIALS

S1. The description of statistical methods

The sign test as a test for comparing two populations can be stated in terms of the probability p that values in one population are greater than values of a second population (paired in some way): $\mathrm{p} := \mathrm{P}(\mathrm{X} > Y)$, where X is the value from the first population and Y is the value from the second population. For random sample of observations we denote every pair (X, Y) where X is greater than Y by a plus sign (+) and every pair (X, Y) where X is less than Y by a minus sign (-). We discard any ties and the number of them is denoted by n_0. In the sign test null hypothesis is that probability p equals 0.5 against alternative hypothesis that probability p is not equal to 0.5. The test statistic is defined as the number of plus signs and its distribution is binomial with n sample size minus the number of ties n_0 and probability $\mathrm{p} = 0.5$ $(\mathrm{B}(\mathrm{n} - \mathrm{n}_0, 0.5))$.

The second test used to compare two populations with paired observations is the Wilcoxon sign-rank test. The Wilcoxon test takes into account the magnitude of differences in n pairs, by considering the absolute values of ranks of these differences. The absolute values of these differences are ranked and then the ranks of positive differences and the ranks of negative differences are summed. In the Wilcoxon test null hypothesis is that the two populations have the same distribution (with the same median). The test statistic T is defined as $\mathrm{T} := \sum \mathrm{R}(+) - \sum \mathrm{R}(-)$, where $\sum \mathrm{R}(+)$ is the sum of ranks of the positive differences and $\sum \mathrm{R}(-)$ is the sum of ranks of the negative differences. The distribution of the test statistic T is based on Fisher's principle of randomization and its normal approximation with mean E(T)=0 and adjusted variance $\mathrm{D}^2_{\mathrm{adj}}(\mathrm{T}) = \frac{\mathrm{n}(\mathrm{n}+1)(2\mathrm{n}+1)}{6}$. Equivalently, a frequently used test statistic for the Wilcoxon sign-rank test is the sum of the positive signed ranks $\mathrm{T}_+ := \sum \mathrm{R}(+)$ with $\mathrm{E}(\mathrm{T}_+) = \frac{\mathrm{n}(\mathrm{n}+1)}{4}$ and $\mathrm{D}^2_{\mathrm{adj}}(\mathrm{T}_+) = \frac{\mathrm{n}(\mathrm{n}+1)(2\mathrm{n}+1)}{24}$.

The Spearman correlation coefficient is defined as the Pearson correlation coefficient between the rank variables

and therefore its interpretation is analogous to that of Pearson's correlation coefficient. In a sample the Spearman correlation coefficient can be calculated by means of following formula:

$$\mathrm{r}_\mathrm{s} = 1 - \frac{6\sum_{\mathrm{i}=1}^{\mathrm{n}} \mathrm{d}_\mathrm{i}}{\mathrm{n} \cdot (\mathrm{n}^2 - 1)},$$

where d_i is the difference between the two ranks of ith observation, and n is the number of sample observations. In statistical test of significance of Spearman rank correlation coefficient the null hypothesis states that "there is no (monotonic) association between the two variables in the population" ($\mathrm{H}_0\colon \rho_\mathrm{s} = 0$ vs. $\mathrm{H}_1\colon \rho_\mathrm{s} \neq 0$, where ρ_s denotes Spearman correlation coefficient in a population).

The test statistic:

$$\mathrm{t} = \mathrm{r}_\mathrm{s}\sqrt{\frac{\mathrm{n} - 2}{1 - \mathrm{r}_\mathrm{s}^2}}$$

under the null hypothesis approximately follows Student's t-distribution with $\mathrm{n} - 2$ degrees of freedom.

Kendall's rank correlation coefficient is commonly referred as Kendall's τ (tau) coefficient. The formula for Kendall's τ_a [1] is following:

$$\tau_a = \frac{S}{N}$$

where Kendall's score S is defined as difference between the number of concordant and the number of discordant pairs, where two pairs $(x_i, y_i), (x_k, y_k)$ are defined as concordant when the product of the difference of their first coordinates and their second coordinates $(x_i - x_k)(y_i - y_k)$ is positive and they are defined as discordant when the product $(x_i - x_k)(y_i - y_k)$ is negative. In the denominator of the formula for τ_a, N denotes the total number of all pairwise combinations which is $n(n-1)/2$ for n ranks' pairs. The value of τ_a is in range $[-1; 1]$ which follows from the fact that the denominator is the total number of pair combinations. When ranks of observations are more similar, τ_a is higher and closer to 1, and for identical ranks τ_a equals 1. But when ranks of observations are more dissimilar τ_a is lower and closer to -1 and for rankings in the oposite order τ_a equalls -1. In the case of independence between variables S and τ_a are close to 0. When there are tied pairs in the data, the coefficient is modified to keep it in the range [−1, 1]. Kendall's coefficient τ_b in contrast to Kendall's τ_a, makes adjustment for ties and its formula is following:

$$\tau_b = \frac{S}{\sqrt{N - n_X}\sqrt{N - n_Y}}$$

where

$$U = \sum_{i=1}^{N_1} u_i(u_i - 1) / 2$$

$$V = \sum_{k=1}^{N_2} v_k(v_k - 1) / 2$$

and where N_1 is the numer of sets of tied x values, u_i is the number of tied x values in the ith set, where N_2 is the numer of sets of tied y values, v_k is the number of tied y values in the kth set [2]. For Kendall's τ, the distribution of the test statistics can be approximated [3] by the normal distribution with mean zero and variance: $\frac{2(2n+5)}{9n(n-1)}$ already for sample sizes greater than 10.

1. Bland, M. *An Introduction to Medical Statistics*, 3rd ed.; Oxford University Press: New York, NY, USA, 2000.
2. Kendall, M.G.; Gibbons, J.D. *Rank Correlation Methods,* 5th ed.; Oxford University Press: New York, NY, USA, 1990.
3. Prokhorov, A.V. Kendall Coefficient of Rank Correlation. In *Encyclopedia of Mathematics*; Hazewinkel, M., Ed.; Springer: Berlin/Heidelberg, Germany, 2001.

S2. The methods of the indicators calculation

Life expectancy (LE)

The LE used in international databases is calculated with the assumption of the constancy of mortality conditions, meaning that the current probability of death for each age is used.

Let q_x denote the probability of dying between age x and x+1.

Starting from an assumed population at birth size 1000, the number of survivors at age x (l_x) is calculated as:

$$l_x = \begin{cases} 1000 & for\ x = 0 \\ l_{x-1} \cdot (1 - q_{x-1}) & for\ x = 1, 2, \ldots \end{cases}$$

and the number of people dying between age x and x+1 as:

$$d_x = l_x \cdot q_x$$

The number of persons living at age x (L_x), understood as person-years at this age is (the coefficient 0.2 was adopted by Eurostat as at the age 0 most deaths occur in the first period of the year; for other ages the coefficient 0.5 is used):

$$L_x = \begin{cases} l_1 + 0.2 \cdot d_0 & for\ x = 0 \\ \dfrac{l_x + l_{x+1}}{2} & for\ x = 1, 2, \ldots \end{cases}$$

The cumulative number of years that a group of persons at age x could expect to live is equal to:

$$T_x = \sum_{i=x}^{K} L_i$$

where K is a age limit adopted or the oldest age for which a value is different than zero.

Looking at T_x construction it is worth noting that mortality rates for older age groups affect more components of the sum than for the younger ones. Additionally, as the mortality is higher in the older age groups, the impact of these mortality rates on the number of years that a group of persons at age x could expect to live is stronger than the impact of younger groups mortality.

Finally life expectancy at age x is calculated as:

$$e_x = \frac{T_x}{l_x}$$

Self-perceived health

Self-perceived general health is assessed asking the question: „How is your health in general? Is it... (1) very good; (2) good; (3) fair; (4) bad; (5) very bad?”. This measure is not based on any comparisons, neither with the health of others, nor with one's own health in the past. Temporary health problems should not be taken into account, the measure refers to health in general. Asking about general health different dimensions of health are included: physical and psychological, but also social and emotional functions.

To confirm the thesis that the health of the older group will affect the SPH value for the total population more and more, because of a growing share of the population 65+ in the total, the simplified simulation for Spain is presented:

Year	Share of the population 65+ in total	SPH very good or good		
		16-64	65+	total population (16+)
2019	19.7%	84.2%	45.8%	76.6%
2050	32.7%	*84.2%*	*45.8%*	*71.6%*

Note: simulated values in italic

There is assumed, that the same percentage of population 16-64 and 65+ will evaluate their health as good or very good in year 2050 as in 2019. The only change is a share of the population 65+ in the total population (it is growing from 19.7% to 32.7% between 2019 and 2050). It can be observed that the value of SPH for total population is 5 percentage points lower in 2050 than in 2019, as impact of the lower indicator value for the population 65+ is stronger.

Health expectancies indicators (HE)

Indicators with a dichotomous valuation of health

The most often used method of HE calculation, including for Eurostat, is Sullivan’s method which is recommended by the WHO. It combines, in an simple manner, two types of information: data regarding mortality (from the life table) and the prevalence of given health dimensions (from the cross-sectional survey). Applying age-specific prevalence to the life table, the total years lived are divided into years spent with and without health problems. HE at a given age represents the number of years expected to live in a given health state, but health may be defined in various ways depending on indicator type. In this method a similar assumption is made as for LE calculation, that the patterns of current prevalence is the same throughout the whole life time.

The **Healthy Life Years (HLY)** indicator presented by Eurostat (also called Disability-Free Life Expectancy) is based on prevalence data regarding general activity limitation, gathered in the EU-SILC survey by asking respondents the question: “For at least 6 months, to what extent have you been limited because of a health problem in activities people usually do? Would you say you have been... (1) severely limited; (2) limited but not severely; (3) not limited at all?” As the self-assessment of health is used here, the survey is conducted on people aged 16 and above. To estimate data for younger groups, results obtained for the group 16-19 is applied to age 15 as well and the prevalence of people younger than 15 is assumed to be half of the prevalence for 16-19.

The prevalence described above is used to divide years expected to live at different ages into years with and without limitations in functioning/disability. To do this the number of person-years at age *x* spent in the health state *j* is calculated:

$$L_x(j) = L_x \cdot \pi_x(j)$$

where L_x is the number of persons living at age x (understood as person-years at this age, as defined above in the section on LE) and $\pi_x(j)$ is the prevalence of a given health state *j* at age *x*.

Then the total number of persons-years expected for people at age *x* to spend in health status *j* is calculated, similar as in LE counting:

$$T_x(j) = \sum_{i=x}^{K} L_i\,(j)$$

Finally the years expected to live in state *j* by a person at age *x*:

$$e_x(j) = \frac{T_x(j)}{l_x}$$

where l_x stands for the number of survivors at age x.

In the HLY indicator presented by Eurostat, a health state without any general activity limitation is adopted as state *j.*

Healthy life expectancy based on self-perceived health (HLE) is constructed for Eurostat purposes using the same, Sullivan's method as HLY. It combines data concerning LE with data on self-perceived health (described above). The answers "very good", "good" and "fair" in the self-perceived health measure are recognized as good health. The prevalence of less than good perceived health is used to divide years expected to live into years spent with and without good perceived health. Analogous estimations for the group younger than 16 are made as for HLY.

Indicator with equivalent years of good health

The indicator **Healthy life expectancy (HALE)** presented by the WHO estimates an average equivalent number of years expected to live in full health. To calculate this equivalent, disability weights are applied to health states.

For a set of disease and injuries *years lost due to disability* (YLD) is estimated, using the Global Burden of Disease study. The YLD as currently used is based on prevalence estimates rather than incidence, calculated without age-weighting and time discounting.

To calculate YLD a disability weight (*DW*) and prevalence (*p*) for each cause have to be assessed. Disability weights reflect the severity of disease/injury outcomes as an effect of the "valuation" of time lived in a non-fatal health state. It is presented as a percentage reduction from full health with a value between 0 and 1. WHO estimations of disability weights are based mostly on GBD analyses, with only some DW having been revised.

The general formula for YLD is:

$$YLD = DW \cdot p$$

The YLD for a disease/injury *i* is calculated as a sum of YLDs related to each condition which is the consequence of this disease or injury (sequelae; *s*):

$$YLD_i = \sum_{s \in S} YLD_s$$

where:

YLD_i – years lost due to disability caused by disease/injury *i*

YLD_s - years lost due to disability caused by sequelae *s*

S – set of sequelae associated with disease/injury *i*

Using the simple sum of YLDs across diseases/injuries may result in a total loss overestimation, even exceeding 100% of the possible time. This can happen especially in older groups of people, as multiple comorbidities very often exist. This is because adjustments for independent comorbidity have been introduced. People with the same health loss are treated in the same way, whether the loss is caused by one or more diseases.

In the case of two comorbid diseases, *1* and *2*, the formula is:

$$YLD_{1+2} = 1 - (1 - YLD_1) \cdot (1 - YLD_2)$$

At this stage, as equivaent years of healthy life lost are estimated (YLD), expected life years at different ages may be divided into years spent in good health equivalent and years lost due to disability. Then Sullivan's method, as described above, can be used to calculate the final HALE value.

Let's denote:

L_x - person-years at age x,

D_x - equivalent lost healthy years fraction at age x

Equivalent years of healthy life lived at age x (YWD_x) is calculated as follows:

$$YWD_x = L_x \cdot (1 - D_x)$$

and finally

$$HALE_x = \frac{\sum_{i=x}^{W} YWD_i}{l_x}$$

where W is the last age interval in the life table.

When abridged life tables are applied, the calculation is done for 5-year intervals, dividing the first 0-4 years interval into groups of age 0 and 1-4 years, with an open-ended interval of 100+ years.

Health gaps indicator: Disability-adjusted life years (DALY)

The DALY measure is an extension of the Potential Years to Life Lost concept, adding a feature regarding time lost due to health problems. One DALY corresponds to one lost year of healthy life. Like the HALE, the DALY applies disability weights to calculate the quantity of life lost due to non-fatal conditions. The DALY for each age-sex group and cause *i* is calculated as the sum:

$$DALY_i = YLL_i + YLD_i$$

where:

YLL_i - years lost due to deaths from the cause of *i*,

YLD_i - years lost due to non-fatal outcomes caused by *i*.

YLL_i is calculated for each age-sex group by multiplying the number of deaths due to cause *i* in a given group by the number of life years lost because of these deaths. The latter is defined by the loss function, representing the maximal life span of a person in good health who receives proper health care services and is not exposed to avoidable health risks or injuries. In the case of WHO calculations this function is based on the highest LEs for the years 2045-50, as projected in the UN World Population Prospects. The loss function specifies maximal LE for each age group separately.

DALY was constructed initially as an incidence-based measure, but since the Global Burden of Disease Study 2010 a prevalence approach has started to be used in YLD calculation. The method of YLD_i calculation is the same as for HALE (as presented above).

S3. The analysis of quotients

Table S1. Descriptive statistics for rank quotients of older sub-population and total population 2010-2019

Indic.	Year	mean	Sd	min	max	Indic.	Year	mean	sd	min	max
LE	*2010*	1.08	0.42	0.25	2.50	HLY	*2010*	1.23	1.08	0.25	5.50
	2011	1.10	0.47	0.20	2.50		*2011*	1.16	0.79	0.19	4.25
	2012	1.12	0.63	0.20	4.00		*2012*	1.13	0.69	0.30	4.00
	2013	1.07	0.38	0.20	2.00		*2013*	1.18	0.81	0.28	4.00
	2014	1.07	0.39	0.22	2.43		*2014*	1.16	0.75	0.31	4.00
	2015	1.06	0.33	0.17	2.00		*2015*	1.13	0.69	0.33	4.00
	2016	1.07	0.39	0.20	2.50		*2016*	1.08	0.45	0.40	2.00
	2017	1.07	0.40	0.22	2.50		*2017*	1.14	0.61	0.31	3.00
	2018	1.07	0.37	0.15	2.11		*2018*	1.10	0.52	0.37	2.61
	2019	1.06	0.33	0.23	2.00		*2019*	1.09	0.45	0.32	2.11
SPH	*2010*	1.09	0.39	0.14	2.00	HALE	*2010*	1.13	0.78	0.25	5.00
	2011	1.08	0.46	0.45	2.33		*2011*	N/A	N/A	N/A	N/A
	2012	1.15	0.76	0.25	4.00		*2012*	N/A	N/A	N/A	N/A
	2013	1.14	0.75	0.50	4.00		*2013*	N/A	N/A	N/A	N/A
	2014	1.12	0.64	0.29	3.40		*2014*	N/A	N/A	N/A	N/A
	2015	1.21	1.14	0.25	7.00		*2015*	1.07	0.47	0.33	3.00
	2016	1.22	1.31	0.44	8.00		*2016*	N/A	N/A	N/A	N/A
	2017	1.18	0.93	0.25	5.50		*2017*	N/A	N/A	N/A	N/A
	2018	1.17	0.91	0.46	5.33		*2018*	N/A	N/A	N/A	N/A
	2019	1.16	0.77	0.36	4.00		*2019*	1.07	0.40	0.25	2.50
HLE	*2010*	1.04	0.32	0.50	2.00	DALY	*2010*	1.29	1.28	0.13	7.00
	2011	1.02	0.19	0.71	1.64		*2011*	1.23	0.98	0.14	5.00
	2012	1.02	0.23	0.71	1.72		*2012*	1.22	0.87	0.13	4.00
	2013	1.03	0.31	0.60	2.17		*2013*	1.17	0.66	0.13	3.00
	2014	1.07	0.53	0.58	3.50		*2014*	1.13	0.55	0.13	2.83
	2015	1.05	0.40	0.50	2.83		*2015*	1.09	0.47	0.22	2.50
	2016	1.06	0.41	0.40	2.60		*2016*	1.09	0.47	0.25	2.50
	2017	1.09	0.61	0.38	4.00		*2017*	1.08	0.44	0.25	2.50
	2018	1.06	0.42	0.50	2.50		*2018*	1.06	0.35	0.38	1.80
	2019	1.05	0.36	0.62	2.20		*2019*	1.06	0.35	0.38	2.00

Note: n denotes number of countries.

Analysis based on LE rankings for 30 countries in the last year (2019) showed that the mean rank quotient for the older sub-population and the total population was 1.06 with an standard deviation from this mean of 0.33. For example, the maximum rank quotient value in 2019 was 2 (for Switzerland, the LE rank for the older population was 3 but for the total population[1] 1.5).

Between 2010 and 2019, the highest average LE rank quotient along with the largest standard deviation (of the LE rank quotient) was observed in 2012. Also in this year the highest maximum of the LE rank quotient occurred, as Iceland ranked 4th in the LE ranking for the older subpopulation and 1st in the LE ranking for the total population.

Between 2010 and 2019, the highest average SPH rank quotient (1.22) along with the largest standard deviation of the SPH rank quotient (1.31) was observed in 2016. In the same year the highest maximum of the SPH rank quotient occurred (8), as Cyprus ranked 16th in the SPH ranking for the older subpopulation and 2nd in the SPH ranking for the total population.

In turn, from 2010 to 2019, the highest mean HLE rank quotient (1.09) along with the largest standard deviation of the HLE rank quotient (0.61) was observed in 2017. The highest maximum of the HLE rank quotient (4) also occurred in 2017, as Italy ranked 12th in the HLE ranking for the older sub-population and 3rd in the HLE ranking for the total population.

The highest mean HLY rank quotient (1.23) along with the largest standard deviation of the HLY rank quotient (1.08) was observed in 2010. Also in 2010 the highest maximum of the HLY rank quotient occurred (5.5), as Malta ranked 5.5 (ex aequo with Switzerland) in the HLY ranking for the older sub-population and first in the HLY ranking for the total population.

The highest mean HALE rank quotient (1.13) along with the largest standard deviation of the HALE rank quotient (0.78) was observed in 2010. Also in this year the highest maximum of the HALE rank quotient occurred, as Iceland ranked 5th in the HALE ranking for the older sub-population and 1st in the HALE ranking for the total population.

During the analyzed period the highest average DALY rank quotient (1.29) along with the largest standard deviation of the DALY rank quotient (1.28) was observed in 2010. Also in 2010 the highest maximum of the DALY rank quotient occurred (7), as Sweden ranked 14 in the DALY ranking for the older sub-population and second in the DALY ranking for the total population.

[1] The first position was taken ex aequo by two countries, Spain and Switzerland (in both cases the LE value for the total population was 84) corresponding to ranks 1 and 2, so these countries were given an average rank of 1.5.

S4. Number of countries with a given size of ranking difference

Table S2. Number of countries with a given size of ranking difference (in absolute values) in the period 2020–2019.

Indicator	Difference size	2010	2011	2012	2013	2014	2015	2016	2017	2018	2019
LE	0	3	1	2	2	2	0	4	4	1	4
	(0, 5]	23	23	24	27	27	30	24	25	28	24
	(5, 10]	4	6	4	1	2	1	3	2	2	0
	more than 10	0	1	0	0	0	0	0	0	0	0
SPH	0	2	3	4	2	7	7	7	4	7	6
	(0, 5]	22	23	21	24	17	15	17	19	13	13
	(5, 10]	7	5	5	4	6	8	6	7	9	9
	more than 10	0	0	1	1	1	1	1	1	2	1
HLE	0	6	9	8	6	9	10	6	5	5	5
	(0, 5]	22	22	22	24	21	19	23	25	24	22
	(5, 10]	3	0	1	1	1	2	2	1	2	2
	more than 10	0	0	0	0	0	0	0	0	0	0
HLY	0	4	6	3	3	1	0	1	4	3	1
	(0, 5]	19	18	21	22	15	22	21	15	16	20
	(5, 10]	5	5	3	3	12	7	7	9	10	6
	more than 10	2	2	3	2	3	2	2	3	2	2
HALE	0	7	*N/A*	*N/A*	*N/A*	*N/A*	5	*N/A*	*N/A*	*N/A*	5
	(0, 5]	22					26				23
	(5, 10]	2					0				3
	more than 10	0					0				0
DALY	0	4	3	3	2	4	4	3	6	6	8
	(0, 5]	20	19	20	23	21	22	22	20	23	21
	(5, 10]	5	8	7	5	5	5	6	5	2	2
	more than 10	2	1	1	1	1	0	0	0	0	0

S5. The graphical presentation of rankings

1. LE

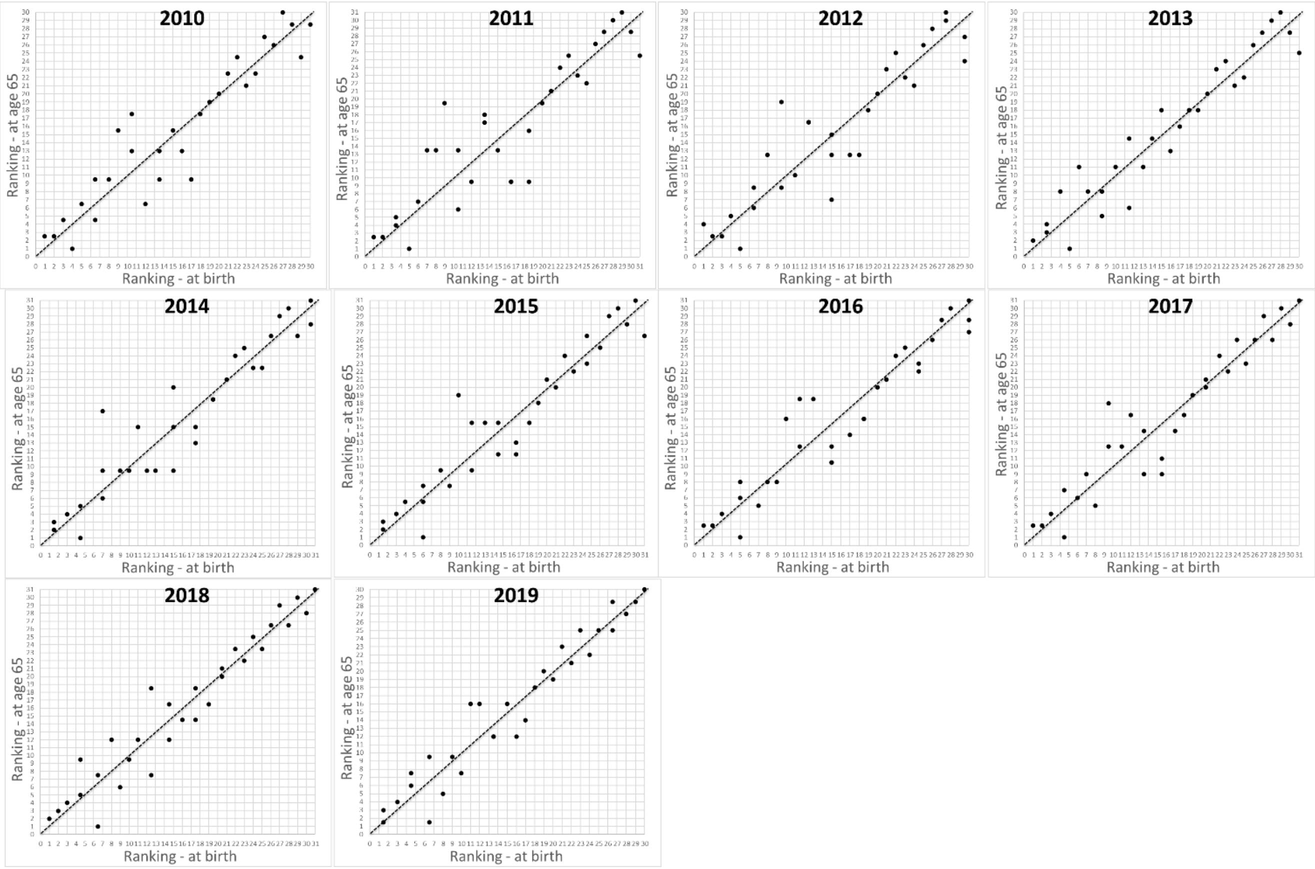

2. SPH

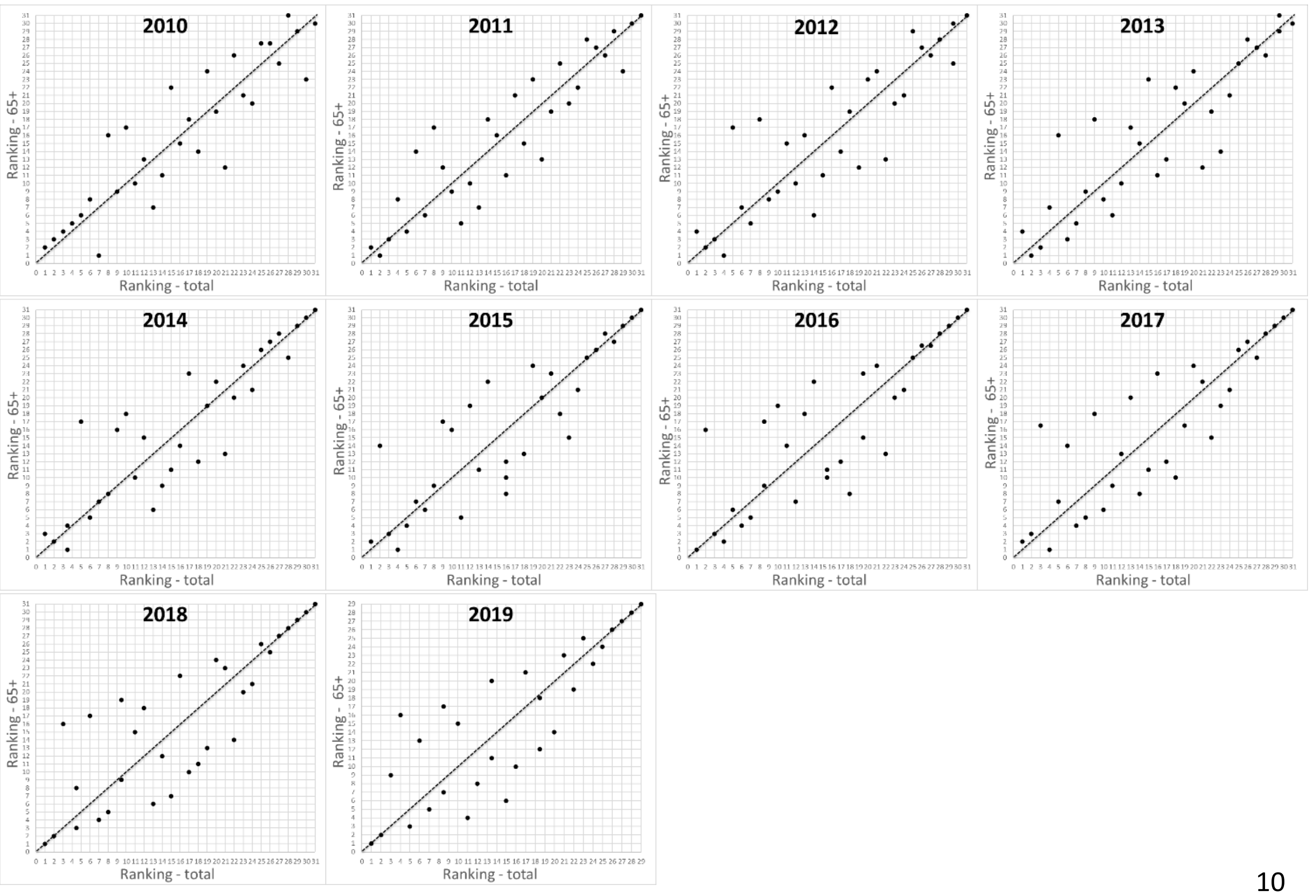

3. HLE

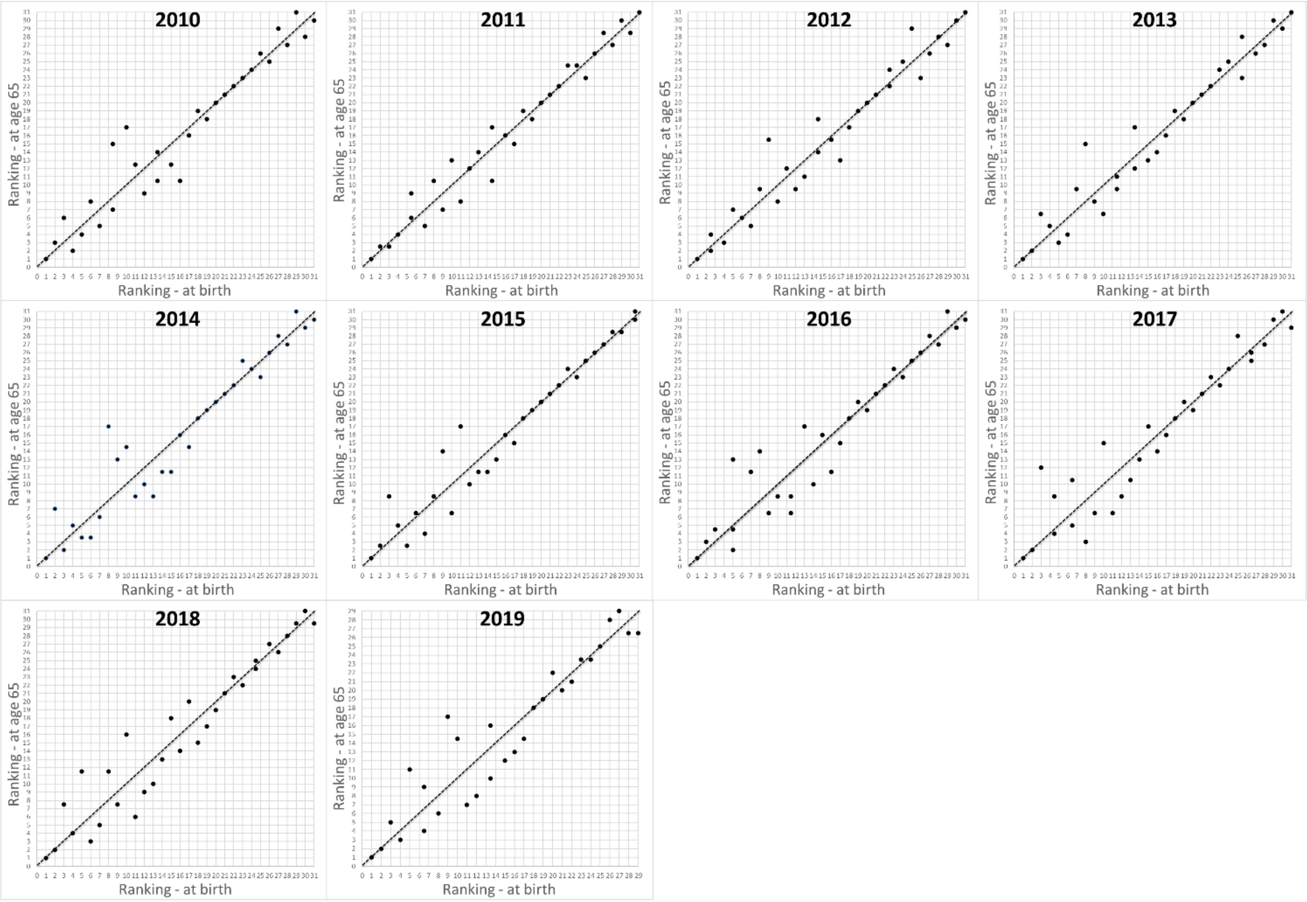
2010
2011
2012
2013
2014
2015
2016
2017
2018
2019
Ranking - at age 65
Ranking - at birth

4. HLY

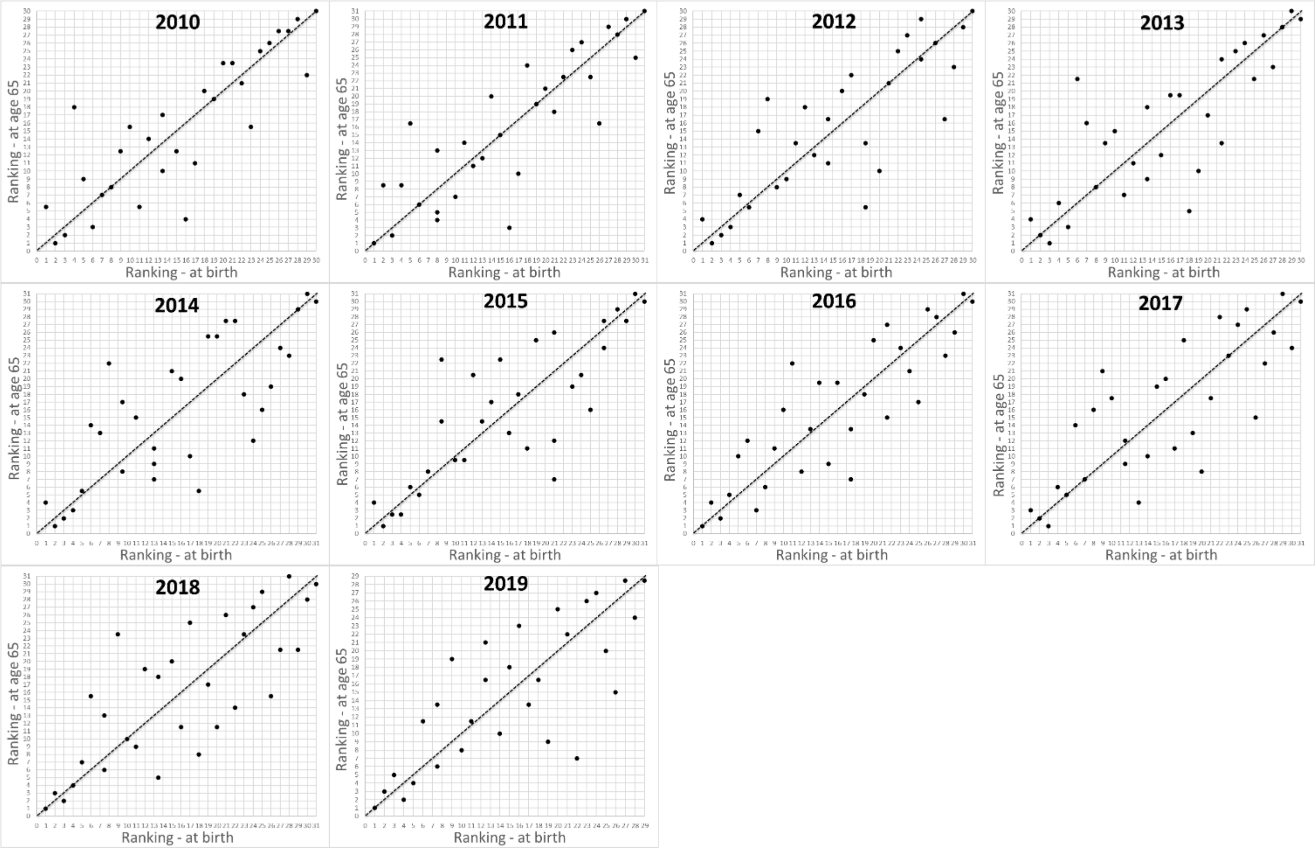
2010
2011
2012
2013
2014
2015
2016
2017
2018
2019
Ranking - at age 65
Ranking - at birth

5. HALE

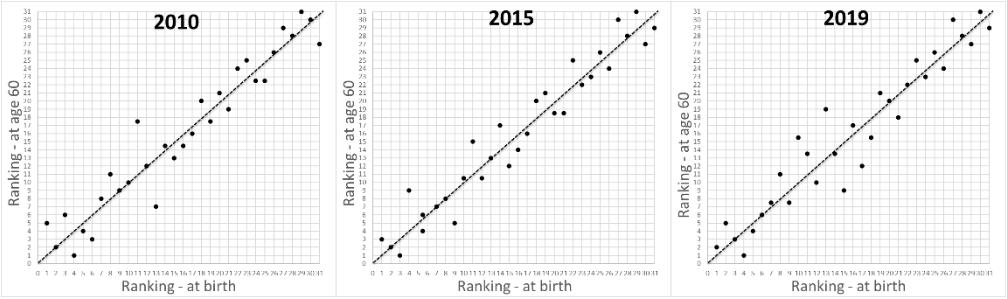
2010
2015
2019
Ranking - at age 60
Ranking - at birth

6. DALY

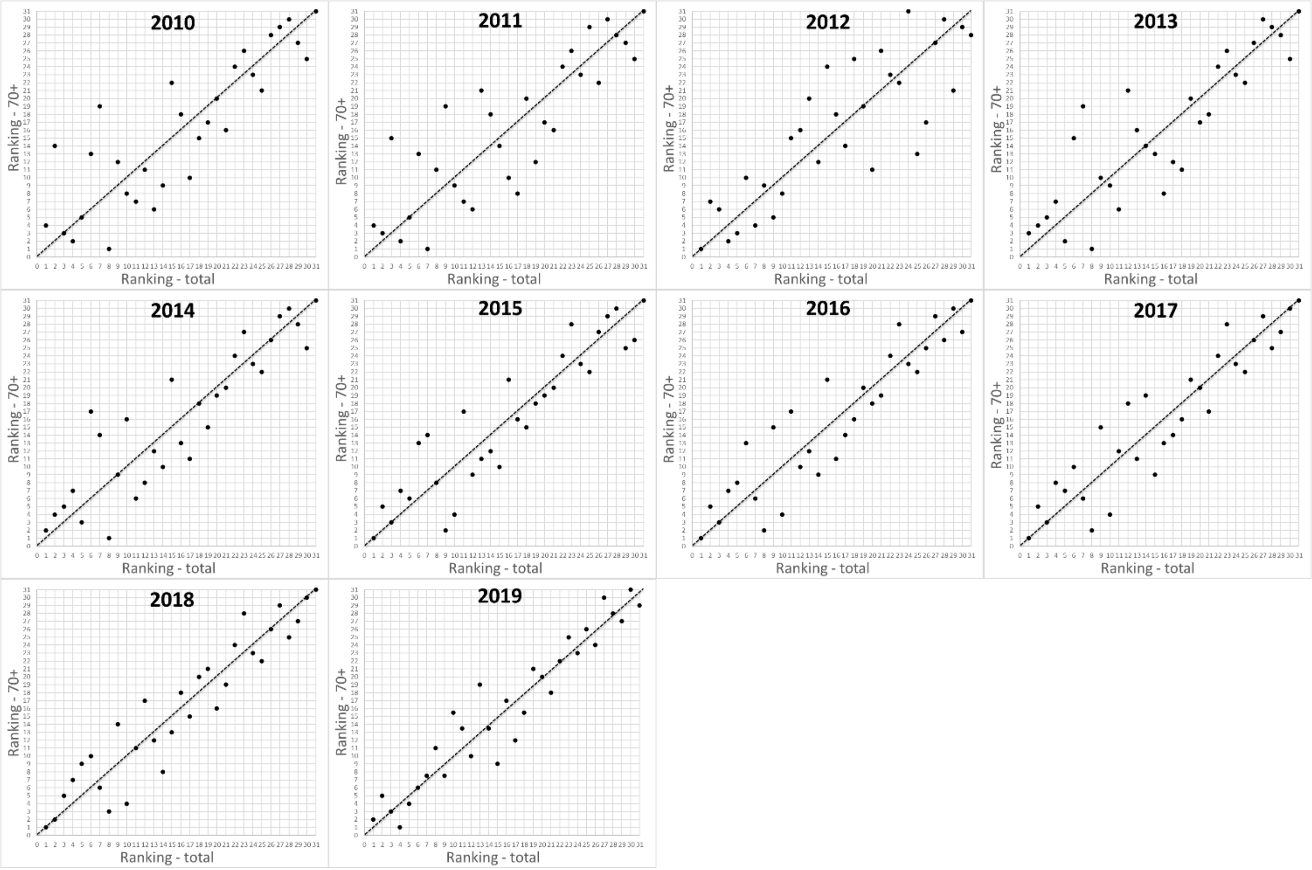
2010
2011
2012
2013
2014
2015
2016
2017
2018
2019
Ranking - 70+
Ranking - total
